# Inhibition of *Porphyromonas gulae* and periodontal disease in dogs by a combination of clindamycin and interferon alpha

**DOI:** 10.1038/s41598-020-59730-9

**Published:** 2020-02-20

**Authors:** Ryota Nomura, Hiroaki Inaba, Hidemi Yasuda, Mitsuyuki Shirai, Yukio Kato, Masaru Murakami, Naoki Iwashita, So Shirahata, Sho Yoshida, Saaya Matayoshi, Junya Yasuda, Nobuaki Arai, Fumitoshi Asai, Michiyo Matsumoto-Nakano, Kazuhiko Nakano

**Affiliations:** 1 Department of Pediatric Dentistry, Osaka University Graduate School of Dentistry, Suita, Osaka Japan; 2 Department of Pediatric Dentistry, Okayama University Graduate School of Medicine, Dentistry and Pharmaceutical Sciences, Okayama, Japan; 3 Yasuda Veterinary Clinic, Meguro, Tokyo, Japan; 4 Department of Pharmacology, School of Veterinary Medicine, Azabu University, Sagamihara, Kanagawa Japan; 5 Department of Veterinary Public Health II, School of Veterinary Medicine, Azabu University, Sagamihara, Kanagawa Japan; 6 Department of Molecular Biology, School of Veterinary Medicine, Azabu University, Sagamihara, Kanagawa Japan; 7 Primo Animal Hospital, Sagamihara, Kanagawa Japan

**Keywords:** Bacteriology, Drug development

## Abstract

*Porphyromonas gulae* is a major periodontal pathogen in dogs, which can be transmitted to their owners. A major virulence factor of *P. gulae* consists of a 41-kDa filamentous appendage (FimA) on the cell surface, which is classified into three genotypes: A, B, and C. Thus far, inhibition of periodontal disease in dogs remains difficult. The present study assessed the inhibitory effects of a combination of clindamycin and interferon alpha (IFN-α) formulation against *P. gulae* and periodontal disease. Growth of *P. gulae* was significantly inhibited by clindamycin; this inhibition had a greater effect on type C *P. gulae* than on type A and B isolates. In contrast, the IFN-α formulation inhibited the expression of IL-1β and COX-2 elicited by type A and B isolates, but not that elicited by type C isolates. Furthermore, periodontal recovery was promoted by the administration of both clindamycin and IFN-α formulation to dogs undergoing periodontal treatment; moreover, this combined treatment reduced the number of FimA genotypes in oral specimens from treated dogs. These results suggest that a combination of clindamycin and IFN-α formulation inhibit *P. gulae* virulence and thus may be effective for the prevention of periodontal disease induced by *P. gulae*.

## Introduction

Periodontal disease is a common infection in dogs^1^, which is characterised by chronic inflammation of periodontal tissue^2^. Periodontal disease affects 44% to 64% of all dogs; this proportion increases to 85% in dogs older than 4 years of age^3,4^. Periodontal disease is caused by the formation of biofilm due to the growth of bacteria in the gingival sulcus^1^. This biofilm elicits an abnormal host immune responses, which is followed by destruction of periodontal tissues such as periodontal ligament and alveolar bone, eventually leading to tooth loss^1,5^.

Among periodontal pathogens, *Porphyromonas gulae* is the bacterial species most often associated with periodontal disease in dogs^6^. FimA (41-kDa fimbriae) is expressed on the cell surface by strains of *P. gulae* bacteria obtained from dogs^7,8^. FimA proteins are divided into three genotypes (A, B, and C), based on differences in their putative amino acid sequences^8^. Among these *fimA* genotypes, type C *P. gulae* is considered to be the most virulent, as it is predominant in the oral cavities of dogs with severe periodontitis^8^. Interestingly, type C *P. gulae* is also prevalent in the oral cavities of dogs with mitral regurgitation^9^, which implies that *P. gulae* may be associated with some types of systemic disease.

Some periodontal pathogens can disrupt the host innate immune response, which results in the exacerbation of periodontal disease^10^. Notably, overexpression of interleukin-1β (IL-1β), cyclooxygenase-2 (COX-2), interleukin-8 (IL-8), and transforming growth factor-β1 (TGF-β1) from gingival cells is closely related to periodontal tissue injury^11–13^. Thus, methods to control the inflammatory response and to eliminate periodontal bacteria are considered to be important for the inhibition of periodontal disease.

Interferon-α (IFN-α), classified as a type I interferon, is generally secreted to combat infection^14^. IFN-α has also been produced as a pharmaceutical agent which is used for treatment of autoimmune and infectious diseases in humans^15^. In addition, a canine IFN-α formulation (InterBerryα^***®***^; Hokusan Co. Ltd., Higashihiroshima, Japan) has been commercially available as a pharmaceutical agent for periodontal treatment in animals since 2014. The administration of canine IFN-α formulation to the oral cavity of dogs has been reported to improve gingivitis symptoms and reduce the number of bacteria in the *Porphyromonas* genus^16^. However, there have been few studies focused on the role of IFN-α in treatment of periodontal disease in dogs.

The recommended approach for prevention and treatment of periodontal disease involves maintenance of oral hygiene by the owner and professional periodontal treatment by veterinarians^1,5^. Antibiotics are generally prescribed in combination with periodontal treatment, such as scaling and root planing, with the aim of reducing the number of pathogenic bacteria^17^. In addition to the use of antibiotics, periodontal recovery relies on the control of inflammatory responses within infected periodontal tissue^18^. Therefore, methods capable of suppressing the host inflammatory immune system should be developed for use in periodontal treatment, in addition to combined application of antibiotic medication and professional periodontal treatment. However, the efficacy of anti-inflammatory treatment of periodontal tissue infected with *P. gulae* has not yet been investigated.

The present study analysed the inhibitory effect of clindamycin, an antibiotic frequently used in periodontal treatment of dogs, on the growth of *P. gulae* strains according to their *fimA* genotypes. In addition, the study investigated whether an IFN-α formulation could inhibit the overexpression of inflammatory responses from gingival epithelial cells induced by *P. gulae* infection. Finally, the study analysed the effects of combined treatment with clindamycin and IFN-α formulation on the periodontal condition and on the levels of *P. gulae* with each *fimA* genotype that were present within oral specimens from dogs.

## Results

### Inhibitory effects of clindamycin on growth of *P. gulae* strains

**Figure 1:**
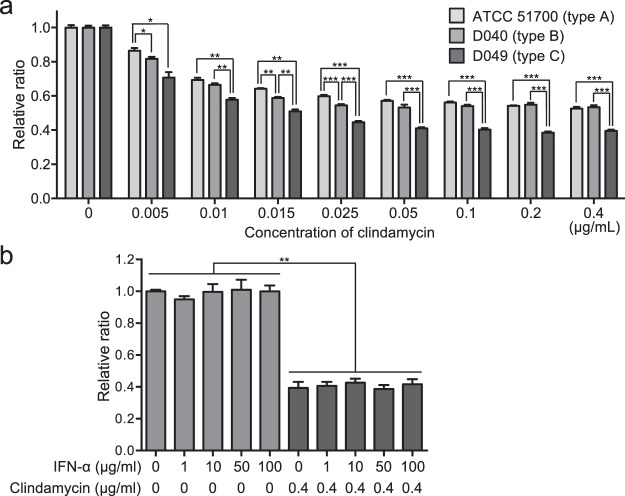
Bacterial growth of *P. gulae* strains. (**a**) Bacterial growth of *P. gulae* strains in the presence of various concentrations of clindamycin. Bacterial growth without clindamycin was defined as the baseline. (**b**) Bacterial growth of *P. gulae* strain D049 in the presence of IFN-α formulation and clindamycin. Bacterial growth without IFN-α formulation and clindamycin was defined as the baseline. Data are shown as the mean ± SD of three independent experiments. There were significant differences as determined by using analysis of variance with Bonferroni correction (*P < 0.05, **P < 0.01, ***P < 0.001).

Clindamycin has been used for the treatment of periodontal disease in dogs^19^. Therefore, we analysed whether clindamycin was effective for *P. gulae* with each *fimA* genotype. Growth of all *P. gulae* strains tested (*fimA* genotypes A, B, and C) was significantly reduced in the presence of more than 0.005% of clindamycin compared with growth without clindamycin (*P* < 0.05) (Fig. 1a). Clindamycin inhibited the bacterial growth of the *P. gulae* strains in a dose-dependent manner. The inhibitory effect on *P. gulae* D049 (type C) was significantly greater than on *P. gulae* ATCC 51700 (type A) and *P. gulae* D040 (type B) in the presence of each concentration of clindamycin (*P* < 0.05). The inhibitory effect on *P. gulae* D040 (type B) was significantly greater than on *P. gulae* ATCC 51700 (type A) in the presence of 0.005%, 0.015%, and 0.025% of clindamycin. When the bacterial growth of *P. gulae* was analysed in the presence of IFN-α formulation, each concentration of the IFN-α formulation showed no effect on the growth of *P. gulae* (Fig. 1b). In addition, the effect of clindamycin on the growth of *P. gulae* was not inhibited by use of the IFN-α formulation.

### Cytokine and enzyme expression in gingival epithelial cells infected with *P. gulae*

**Figure 2:**
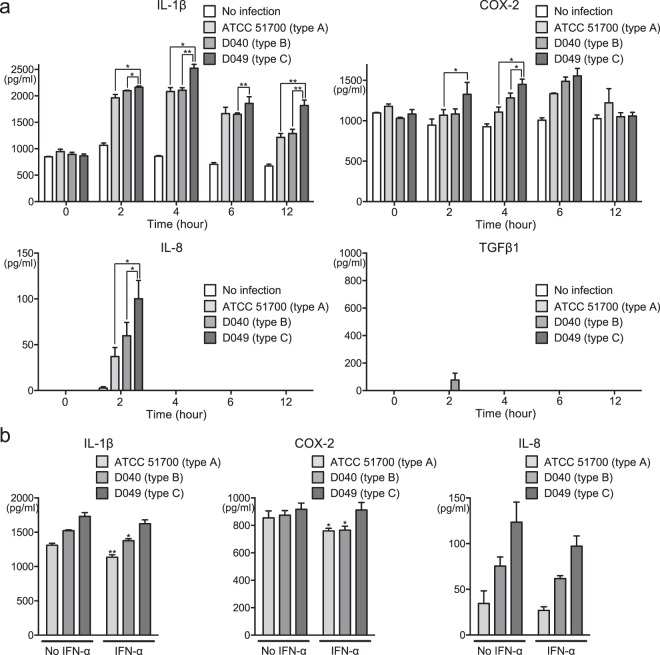
Production of cytokines or COX-2 enzyme from Ca9-22 cells infected with *P. gulae* strains. (**a**) Amounts of cytokine or enzyme production at multiple time points. Data are shown as the mean ± SD of three independent experiments. There were significant differences in cytokine and enzyme production, as determined by using analysis of variance with Bonferroni correction (*P < 0.05 and **P < 0.01). (**b**) Amounts of cytokine or enzyme production in the presence of IFN-α formulation. Data are shown as the mean ± SD of three independent experiments. There were significant differences in cytokine and enzyme production, relative to cells that did not receive the IFN-α formulation, upon infection with each *P. gulae* strain, as determined by using analysis of variance with Bonferroni correction (*P < 0.05).

*P. gingivalis*, a species closely related to *P. gulae*, induces inflammatory disorders which cause aggravation of periodontal disease^20^. Gingival epithelial cells play an important role in preventing bacterial invasion deeper into tissue^21^; however, changes in the host inflammatory response produced by gingival epithelial cells exposed to *P. gulae* remain unknown. The relative ratios of mRNA expression levels of IL-1β, COX-2, IL-8, and TGF-β1 in Ca9-22 human gingival epithelial cells infected with *P. gulae* strains were analysed with their respective levels at 0 h after *P. gulae* infection defined as 1.0. IL-1β, COX-2, and IL-8 levels in the presence of each *P. gulae* strain were highest at 2 h after infection; these levels were significantly higher than those in uninfected cells (*P < *0.001) (Supplementary Fig. 1a). In addition, at 2 h after infection, IL-1β expression induced by *P. gulae* D049 (type C) infection was significantly higher than that induced by *P. gulae* ATCC 51700 (type A) or *P. gulae* D040 (type B) (*P* < 0.05); a similar trend was observed with respect to COX-2. IL-8 expression tended to be higher in the presence of strain D049 (type C) than in the presence of strains ATCC 51700 (type A) or D040 (type B); however, there were no significant differences among the strains. There was no change in the expression level of TGF-β1 at any time after *P. gulae* infection. Subsequently, we measured the protein levels of IL-1β, COX-2, IL-8, and TGF-β1 in *P. gulae*-infected Ca9-22 cells (Fig. 2a). In Ca9-22 cells infected with each strain of bacteria, increased protein levels of IL-1β, COX-2, and IL-8 were observed, whereas protein expression of TGF-β1 was nearly absent, regardless of the presence of the bacteria. Similar to the mRNA analysis, expression levels of IL-1β, COX-2, and IL-8 induced by strain D049 (type C) infection were significantly higher than expression levels induced by strains ATCC 51700 (type A) or D040 (type B).

IFN-α has been reported to be effective in chronic and infectious diseases^22^, and oral administration of an IFN-α formulation improved gingival inflammation in dogs with periodontal disease^16^. Therefore, each *P. gulae* strain and an IFN-α formulation were simultaneously added to respective cultures of Ca9-22 cells, and the expression levels of IL-1β, COX-2, and IL-8 were analysed 2 h after incubation. Before the experiment, the IFN-α formulation, clindamycin, and combined treatment with the IFN-α formulation and clindamycin were confirmed to have no effect on the growth of uninfected Ca9-22 cells (Supplementary Fig. 2a,b). Treatment with the IFN-α formulation significantly reduced the mRNA expression levels and protein production levels of IL-1β and COX-2 in Ca9-22 cells that were infected with *P. gulae* ATCC 51700 (type A) and *P. gulae* D040 (type B), respectively (*P* < 0.05); no changes in IL-8 expression were observed (Supplementary Fig. 1b and Fig. 2b). In addition, such changes in expression levels were not observed in Ca9-22 cells that were infected with *P. gulae* D049 (type C).

### Changes in periodontal conditions before and after clinical treatment

**Figure 3:**
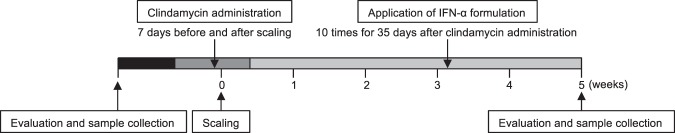
Schedule of periodontal treatment of dogs in the present study, using clindamycin and IFN-α formulation.

**Figure 4:**
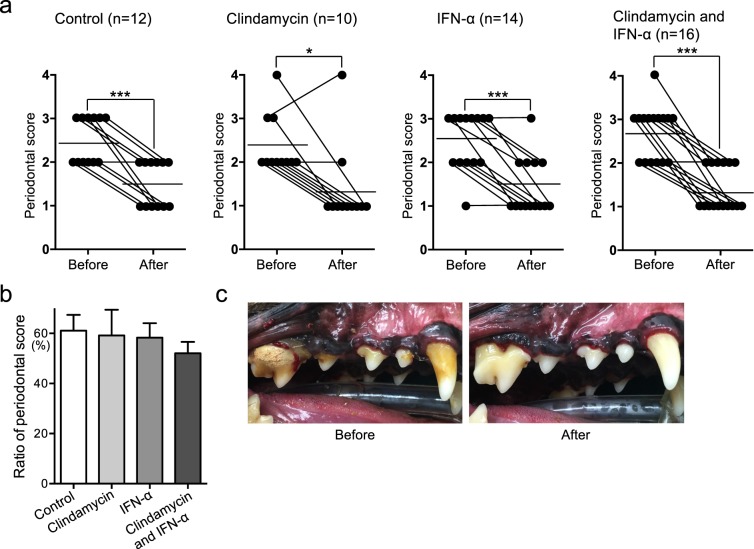
Periodontal conditions before and after clinical treatment. (**a**) Periodontal scores before and after treatment. There were significant differences as determined by using analysis of variance with Bonferroni correction (*P < 0.05, ***P < 0.001). (**b**) Ratio of periodontal scores after treatment relative to those before treatment. (**c**) Representative images of periodontal condition before and after treatment with clindamycin and IFN-α formulation.

The schedule of treatment is summarized in Fig. 3. Fifty-two dogs were divided into four groups with or without pharmaceutical treatments, as follows: no pharmaceutical treatment (control group; n = 12), clindamycin treatment (clindamycin group; n = 10), IFN-α formulation treatment (IFN-α group; n = 14), and clindamycin and IFN-α formulation combined treatment (combination group; n = 16) (Table 1). In the clindamycin group, clindamycin was ingested for a total of 7 days (4 days before and 3 days after scaling). In the IFN-α group, an IFN-α formulation (InterBerryα^***®***^; Hokusan Co. Ltd.) was applied to the gingival margin of all teeth, 10 times total over 35 days. Gingival and periodontal scores of dogs were assessed at the beginning and end of clinical treatment, in accordance with previously described methods^23^. Periodontal scores in all groups after the treatment were significantly lower than those before treatment (*P* < 0.05) (Fig. 4a). In addition, the relative ratios were calculated for periodontal scores after periodontal treatment compared with those before periodontal treatment; the combination group had the lowest relative ratio, although there were no significant differences among groups (Fig. 4b). As shown in Fig. 4c, accumulations of dental plaque and dental calculus around teeth were reduced and gingival conditions were improved after periodontal treatment in the combined group.

**Table 1:** Dog breeds included in the present study.

Prescription	Breeds	Age	Sex	Weight	*fimA* genotype	
					pre	post
Control (n = 12)	Cavalier King Charles Spaniel	10Y6M	Female	10.1	B	A
	Miniature Dachshund	9Y11M	Male	6	(—)	A
	Miniature Dachshund	9Y4M	Female	5.3	A	A
	Miniature Dachshund	12Y6M	Female	5.5	A	A
	Yorkshire Terrier	13Y8M	Female (spay)	2.7	A	A
	Corgi	8Y0M	Male	12.3	B, C	B, C
	Chihuahua	10Y3M	Male (castration)	2.8	(—)	A
	Toy Poodle	8Y0M	Male (castration)	3.4	A	A
	Mix	1Y5M	Male	3	A	A
	Miniature Dachshund	14Y0M	Female (spay)	4.6	A	B, C
	Toy Poodle	3Y7M	Male	3.2	A, B, C	C
	Miniature Dachshund	13Y6M	Male (castration)	6.9	B	B
Clindamycin (n = 10)	Toy Poodle	13Y5M	Male	5.4	A, C	A
	English Cocker Spaniel	11Y7M	Female (spay)	10.8	A, C	C
	Toy Manchester	9Y0M	Male (castration)	4.3	C	(—)
	Mix	13Y0M	Female	5.5	C	B, C
	Italian Greyhound	10Y2M	Female (spay)	5.1	A, B, C	A
	Yorkshire Terrier	9Y8M	Male (castration)	1.2	A, C	(—)
	Shiba	7Y10M	Female (spay)	7.5	A, C	A
	Pomeranian	14Y3M	Male (castration)	4.1	B	B
	Papillon	13Y0M	Female (spay)	3.3	Untypeable	B
	Toy Poodle	12Y1M	Male	8.3	A, C	A, C
IFN-α (n = 14)	Miniature Dachshund	10Y6M	Female (spay)	5.9	A	(—)
	Shetland Sheepdog	10Y7M	Male	11.3	C	(—)
	Miniature Dachshund	11Y11M	Male	5.1	A	A
	Miniature Dachshund	9Y10M	Male	4.3	A	A
	Mix	12Y0M	Female	4.1	A	(—)
	Miniature Dachshund	7Y1M	Female (spay)	5.7	A	(—)
	Cavalier King Charles Spaniel	5Y2M	Male (castration)	9.8	A	A
	Toy Poodle	9Y7M	Female	5.5	A, B, C	C
	American Cocker Spaniel	12Y1M	Female	7.5	A, C	A
	Miniature Dachshund	15Y10M	Female (spay)	2.9	A, B, C	B, C
	Miniature Dachshund	15Y4M	Female (spay)	4.1	A, B, C	A, B, C
	Miniature Schnauzer	4Y8M	Male (castration)	5.8	C	C
	Toy Poodle	9Y10M	Female (spay)	3.8	A	A
	Chihuahua	9Y10M	Male	2.7	A	A
Clindamycin and IFN-α (n = 16)	Japanese Terrier	11Y8M	Female	13.4	B	(—)
	Yorkshire Terrier	11Y9M	Male (castration)	2.8	A, C	A
	Miniature Dachshund	13Y9M	Male (castration)	4.3	A, B, C	B
	Toy Poodle	8Y8M	Male (castration)	2.8	B	(—)
	Petit Basset Griffon Vendeen	7Y11M	Male	12.6	C	B, C
	Pomeranian	11Y5M	Male	4.4	B, C	(—)
	Bearded Collie	11Y11M	Female	16.7	A, C	A
	Toy Poodle	6Y9M	Male	3	B, C	(—)
	Toy Poodle	7Y5M	Male (castration)	6.7	A, B, C	A
	Toy Poodle	7Y11M	Male (castration)	5.4	C	(—)
	Toy Poodle	13Y0M	Male (castration)	4.6	B	B
	Toy Poodle	15Y2M	Female (spay)	4.9	C	Untypeable
	Miniature Poodle	10Y0M	Female	6.4	B	(—)
	Pug	7Y0M	Female	8.7	A, B, C	A, C
	Toy Poodle	10Y10M	Male (castration)	3.4	Untypeable	Untypeable
	Miniature Dachshund	13Y11M	Female (spay)	6.6	A	A

### *P. gulae* detection before and after clinical treatment

**Figure 5:**
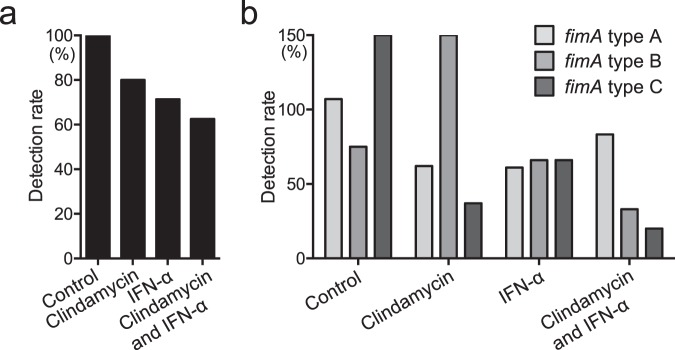
Rates of *P. gulae* detection before and after clinical treatment. (**a**) Rates of *P. gulae* detection after treatment, relative to those before treatment. (**b**) Rates of detection of *fimA* genotypes after treatment, relative to those before treatment.

Oral swab specimens were collected from the gingival margin of the maxillary right or left canine and fourth maxillary premolar at the beginning and end of clinical treatment, as described previously^24^. Distributions of *P. gulae* and *fimA* genotype classification in the oral specimens were determined using polymerase chain reaction (PCR)-based methods, as previously described^8,25^. With the exception of two dogs in the control group, all dogs were positive for *P. gulae* before periodontal treatment; all *P. gulae*-positive dogs in the control group were positive for *P. gulae* after treatment (Table 1, Fig. 5a). Two of 10 dogs (20%) in the clindamycin group and four of 14 dogs (28.6%) in the IFN-α group were negative for *P. gulae* after treatment. The combination group had the lowest rate of *P. gulae* detection after treatment: *P. gulae* was absent from the oral cavity in six of 16 dogs (37.5%). Next, we determined the relative ratio of the detection of each *fimA* type after treatment, compared with that before treatment. In the control group, only the relative ratio of *fimA* type B was reduced after treatment (Fig. 5b). In contrast, the relative ratios of *fimA* types A and C were reduced after treatment in the clindamycin group; the relative ratios of all *fimA* genotypes were reduced after treatment in the IFN-α and combination groups. Notably, the relative ratios of *fimA* type C were dramatically reduced after treatment, which were present in 37.5% and 20% of dogs in the clindamycin and combination groups, respectively. When the detection rates of *fimA* genotypes in each group were compared with those in the control group, detection rates of all *fimA* genotypes in each group (with the exception of *fimA* type B in the clindamycin group) were lower than those in the control group.

## Discussion

Periodontal disease is an inflammatory disease caused by bacterial infection^5^. Bacterial plaque remains at the gingival sulcus between the teeth and gingiva, resulting in gingivitis where the margin of the gingiva becomes red and swollen^5^. The presence of chronic gingivitis results in the formation of irreversible deep periodontal pockets at the gingival sulcus^5^. Consequently, alveolar bone resorption and tooth mobility occur, followed by loss of teeth in some instances^26^. Periodontal disease is present in more than 80% of mature dogs and is one of the most prevalent diseases in dogs^26^. *P. gulae* is a major periodontopathic bacteria that has been strongly associated with the deterioration of periodontal disease in dogs^7^. However, there have been no studies of the potential for preventing periodontal disease in dogs by reducing the pathogenicity of *P. gulae*. In the present study, we examined the inhibition of *P. gulae* and periodontal disease by using treatment with clindamycin and IFN-α formulation.

Antibacterial agents are often prescribed for a period of 1 week in combination with periodontal treatment; this approach is useful for reduction of periodontal bacteria^17^. Among antibiotics prescribed during scaling and root planing treatment of dogs, the lincomycin-derived antibacterial agent clindamycin is widely used for prevention of infection^27^. In the present study, 0.005–0.4 μg/ml clindamycin was used to assess the inhibitory effect of antibiotic treatment on growth of *P. gulae*. These low concentrations of clindamycin could inhibit the growth of all *fimA* types of *P. gulae*, among which the inhibitory effect most prominently affected the type C *P. gulae* strain. In the clinical portion of this study, type C *P. gulae* was also reduced in dogs that received clindamycin treatment. In our previous study, type C *P. gulae* was frequently detected in dogs with periodontitis, at rates significantly higher than those in healthy dogs^8^; therefore, clindamycin may be especially effective for dogs with severe periodontitis.

The Ca9-22 cell line was used as an *in vitro* counterpart of gingival epithelial cells^28^. Bacterial components or chemical regents have previously been reported to induce several inflammatory responses, such as IL-1β, COX-2, and IL-8, in Ca9-22 cells^29,30^. In addition, the mRNA expression levels of TGF-β1 in several cell lines were reportedly upregulated by exposure to virus protease, although the level of TGF-β1 mRNA in Ca9-22 was unchanged^31^. Thus, we examined the effects of *P. gulae* infection on IL-1β, IL-8, COX-2, and TGF-β1.

IL-1β has rapidly emerged as a key player in the regulation of inflammatory processes, which is capable of augmenting IL-8 production^32,33^. In addition, IL-1β exhibits an important role in the modulation of other inflammatory cytokines in human gingival epithelial cells infected with *P. gingivalis*^34^. COX-2 is the key enzyme involved in prostaglandin synthesis, and is expressed in inflammatory cells^35^; it is also known as a potent stimulator of bone resorption and is associated with periodontal attachment loss^36^. IL-8 is a key player in inflammatory conditions with a potent neutrophil recruiting and activating capacity^32^; it is induced in gingival epithelial cells upon exposure to several types of periodontopathic bacteria^37^. TGF-β1 has been shown to play a critical role in anti-inflammatory signaling^38^, which occurs in wound healing and periodontal regeneration^11^. Our results indicated that *P. gulae* infection induced overexpression of IL-1β, IL-8, and COX-2, potentially leading to inflammation involved in deterioration of periodontal disease.

Several oral pathogens, such as *Fusobacterium nucleatum*, *Streptococcus sanguinis*, and *Aggregatibacter actinomycetemcomitans*, have been reported to induce increased mRNA expression levels of IL-1β, IL-8, and tumour necrosis factor-α in Ca9-22 cells^29,37^. Stimulation of immortalised human gingival epithelial cells with vesicles from *P. gingivalis* bacteria led to upregulation of COX-2 and IL-8 mRNA^36^. Additionally, stimulation with *P. gulae* has been shown to induce increased secretion of IL-1β^39^. In the present study, we found that secretion and expression levels of IL-1β, IL-8, and tumour necrosis factor-α were increased upon infection with *P. gulae*. These findings suggest that *P. gulae* infection induces mRNA expression of a wide variety of inflammatory-related proteins in Ca9-22 cells. TGF-β1 mRNA and protein expression were reportedly upregulated by infection with bacteria such as *Staphylococcus aureus*, *Helicobacter pylori*, and group A *Streptococcus*^38,40,41^. In contrast, infection of human gingival fibroblast cells with *P. gingivalis* resulted in an increase in mRNA expression levels of TGF-β1, but did not influence the corresponding protein expression levels^42^. Following *P. gulae* infection in the present study, TGF-β1 mRNA expression levels were increased, while corresponding protein expression levels were not affected; this suggested that the effects of *P. gulae* on TGF-β1 expression were similar to those of *P. gingivalis*. Moreover, these findings indicate that the protein expression levels of TGF-β1 may not always reflect its mRNA expression levels.

Pharmaceutical agents containing IFN-α are widely used as antiviral and anticancer agents, because IFN-α can inhibit the growth of viruses and cancer cells^43^. In addition to these effects, IFN-α can regulate immune and inflammatory processes^44^. Therefore, we examined whether a canine IFN-α formulation could suppress inflammatory responses in gingival epithelial cells that had been infected with *P. gulae*. We found that the IFN-α formulation could reduce the expression of IL-1β and COX-2, which were upregulated by infection with *fimA* types A and B *P. gulae* strains. Furthermore, we found that clindamycin was more effective than the IFN-α formulation against the *fimA* type C *P. gulae* strain; thus, combined use of an IFN-α formulation and clindamycin may be effective for *P. gulae* strains, regardless of *fimA* genotype. In future studies, we plan to analyse effects of the combined use of an IFN-α formulation and clindamycin on the expression of other cytokines and enzymes, as well as on the survival of various periodontopathogenic bacteria.

In the present study, we examined whether an IFN-α formulation is useful for the suppression of inflammatory responses induced by *P. gulae* infection and whether it may be effective for treatment of periodontal disease in dogs. We applied the IFN-α formulation after removal of dental plaque and dental calculus to allow the IFN-α formulation to penetrate into the periodontal tissues. In addition, we continued the application of the IFN-α formulation for 35 days to suppress chronic inflammation, because most periodontal diseases are chronic diseases^45^. Although the IFN-α formulation was applied to all dogs for the same duration in the present study, the duration and frequency at which the IFN-α formulation is applied should be determined on the basis of periodontal disease severity in future studies.

The IFN-α formulation can be used in all dogs regardless of age, based on its approval by the Ministry of Agriculture, Forestry and Fisheries. The manufacturer currently recommends that the IFN-α formulation is administered to dogs with a periodontal score of ≤1. However, we have successfully shown that the IFN-α formulation is also effective in dogs with more severe periodontal conditions, which indicates that the IFN-α formulation can be used for treatment of dogs with more severe periodontitis. During the experimental period, no dogs had any problems with gingival or physical conditions due to the administration of the IFN-α formulation. Based on our results, the IFN-α formulation could be used in the treatment of dogs at various ages and with various degrees of periodontal disease.

In the present study, the periodontal score was reduced in all dogs after clinical periodontal treatment. These reductions in periodontal scores were also observed in the control group, which serves as evidence for the importance of mechanical removal of dental plaque and dental calculus by scaling or root planing, consistent with the findings of a previous report^46^. However, the group that received combined treatment showed the lowest periodontal score among all groups; therefore, combined use of clindamycin and IFN-α formulation may be effective as a supporting method for the improvement of periodontal condition, following mechanical cleaning.

In humans, no IFN-α formulation has been used in periodontal treatment. However, it may be useful to investigate whether combined treatment with antibiotics and an IFN-α formulation can be effective in human periodontal disease, because dog owners sometimes are colonized by *P. gulae* from their dogs^47^. In addition, it may be also informative to analyse whether combined treatment with antibiotics and an IFN-α formulation is effective to inhibit *P. gingivalis* growth, because this bacterial species is detected in both humans and dogs with periodontal disease.

To the best of our knowledge, this is the first study to show that the combination of clindamycin and IFN-α formulation is effective in improving the periodontal condition in dogs. In this study, we showed that combination therapy with clindamycin and IFN-α could improve periodontal conditions and reduce *P. gulae* in randomly selected dogs. However, in addition to the effects of clindamycin and IFN-α, genetic and environmental factors may have influenced our findings, because only a small number of dogs participated in this study. Therefore, larger clinical studies are needed using more dogs with different backgrounds.

In summary, clindamycin is an effective antibiotic for inhibition of the growth of *P. gulae*, and the application of an IFN-α formulation can suppress inflammatory responses produced from *P. gulae*-infected gingival epithelial cells. The combination of clindamycin and IFN-α formulation in the treatment of canine periodontal disease may contribute to improved periodontal conditions in dogs.

## Methods

### Bacterial strains and cell cultures

*P. gulae* strains ATCC 51700 (*fimA* type A), D040 (*fimA* type B), and D049 (*fimA* type C) were selected from the stock culture collection in our laboratory^7,8,24^. Bacterial cells were grown anaerobically at 37 °C for 24 h in trypticase soy broth supplemented with yeast extract (1 mg/ml), haemin (5 μg/ml), and menadione (1 μg/ml), as previously described^48^; they were then used in the following experiments. Ca9-22 cells (originally isolated from human gingival epithelia) were obtained from the Japanese Collection of Research Bioresources (Tokyo, Japan); these cells were used as an *in vitro* counterpart of gingival epithelial cells^28^ because they have been widely used as an *in vitro* culture model of gingival epithelial cells^28,49^. The cells were cultured in Dulbecco’s modified Eagle’s medium (DMEM) (Wako, Osaka, Japan) supplemented with 10% fetal bovine serum at 37 °C in 5% CO_2_.

### Bacterial growth

Bacterial growth was analysed in accordance with previously described methods, with some modifications^50–52^. Various concentrations of clindamycin were tested for their effects on *P. gulae* growth: 0.005, 0.01, 0.015, 0.025, 0.05, 0.1, 0.2, and 0.4 μg/ml. In addition, various concentrations of IFN-α formulation were tested: 0, 1, 10, 50, and 100 μg/ml. Clindamycin and IFN-α formulation, separately or in combination, were added to the trypticase soy broth supplemented with yeast extract, haemin, and menadione used for bacterial suspension growth. In addition, overnight cultured *P. gulae* bacteria were added to the media at a density of 4 × 10^8^ CFU/ml and then cultured at 37 °C for 24 h. Bacterial growth after incubation was measured by determining the optical density at 600 nm using a microplate reader (SH-1000 Lab, Corona Electric, Katsuta, Japan), because the number of *P. gulae* bacteria in a given suspension has been previously estimated by measurement of the optical density at 600 nm and subsequent extrapolation from a standard curve^53^. The relative ratio of growth of each *P. gulae* strain was calculated by comparison with the optical density at 600 nm value of each bacterial broth without clindamycin. All assays were performed in triplicate on three separate occasions (n = 9).

### Real-time reverse transcription-polymerase chain reaction (RT-PCR) for quantitative detection of mRNA expression

Ca9-22 cells were incubated in DMEM with 10% fetal bovine serum until confluent. After incubation, the monolayers of Ca9-22 cells were washed three times with serum-free DMEM to remove unattached cells. Additionally, the number of viable cells in each monolayer was determined using trypan blue dye exclusion and cell counting method. IFN-α (100 μg/ml) was preincubated with Ca9-22 cells prior to addition of bacteria. Overnight cultured *P. gulae* were harvested and washed with sterile phosphate-buffered saline. The bacteria were then diluted to 1 × 10^8^ CFU/ml in DMEM and used to infect Ca9-22 cells. For experiments involving bacterial infection of Ca-22 cells, we used the concept of multiplicity of infection (MOI), which is commonly defined as the ratio of infectious microorganisms to cells in a culture^54^. Ca9-22 cells were diluted to a density of 1 × 10^6^ and infected with 1 × 10^8^ CFU of respective *P. gulae* strains at MOI of 100 for 0–12 h. Then, total RNA was extracted from these *P. gulae*-infected Ca9-22 cells and cDNA was synthesized as described previously^55^. Briefly, total RNA from Ca9-22 cells was isolated using TRIsure (BIOLINE, Luckenwalde, Germany) and converted into cDNA using an iScript™ cDNA Synthesis kit (Bio-Rad, Hercules, CA, USA) in accordance with the manufacturer’s instructions. cDNAs were amplified using a QuantiFast SYBR Green PCR master mix (Qiagen, Valencia, CA, USA), in accordance with the manufacturer’s instructions. The primers specific for genes encoding IL-1β, COX-2, IL-8, and TGF-β1, which were used in this study, are listed in Table 2. GAPDH was used as a housekeeping control and negative reverse transcription reactions were included in each assay. Expression values for mRNA were quantified by the ΔΔCt method, using GAPDH as the control. All assays were performed in triplicate on three separate occasions (n = 9).

**Table 2:** Reverse transcription polymerase chain reaction primers used in the present study.

Specific primer set	Sequence (5′-3′)	Size (bp)	References
GAPDH	GTCTTCACCACCATGGAGAAG	210	^58^
	GTTGTCATGGATGACCTTGGC		
IL-1β	CTTTGAAGCTGATGGCCCTAAA	101	^59^
	AGTGGTGGTCGGAGATTCGT		
COX-2	CACAGGCTTCCATTGACCAGA	240	^36^
	GTGCTCCAACTTCTACCATGG		
IL-8	CTTGGCAGCCTTCCTGATTTC	209	^36^
	CCAGACAGAGCTCTCTTCCAT		
TGF-β1	CACCCGCGTGCTAATGGT	100	^60^
	CTCGGAGCTCTGATGTGTTGAA		

### Enzyme-linked immunosorbent assays (ELISAs)

*P. gulae* strains were used to infect Ca9-22 cells with or without administration of the IFN-α formulation, as described in the above section regarding RT-PCR. ELISAs were performed in accordance with previously described methods, with some modifications^56^. Ca9-22 cells were stimulated with *P. gulae* strains in the presence or absence of IFN-α for 24 h. For the quantification of IL-1β, COX-2, IL-8, and TGF-β1 in cell lysate at each time point, sandwich ELISAs were performed using the Human IL-1β ELISA kit (Proteintech Group Inc., Rosemont, IL, USA), Human/mouse total COX-2 DuoSet IC ELISA (R&D Systems Inc., Minneapolis, MN, USA), IL-8 ELISA kit (Proteintech Group Inc.) and TGF-β1 ELISA kit (Proteintech Group Inc.), respectively, in accordance with the manufacturers’ instructions. Absorbance was measured at 450 nm, with correction to 550 nm, using a SH-1000 Lab microplate reader (Corona Electric, Ibaraki, Japan). All assays were performed in triplicate on three separate occasions (n = 9).

### Schedule in clinical experiment

The clinical experiment was conducted in full adherence to the Declaration of Helsinki. All study protocols were approved by the Animal Research Committee of Azabu University. Prior to the clinical experiment, all owners were informed of the content of the study and gave written informed consent for approval of their pets’ participation. The schedule for clinical treatment is summarized in Fig. 3. In total, 52 dogs (28 males, 24 females; median age: 10 years [range: 1–15 years]) were enrolled (Table 1); all dogs received periodontal treatment under general anaesthesia. The subjects were divided into four groups with or without pharmaceutical treatments, as follows: no pharmaceutical treatment (control group; n = 12), clindamycin treatment (clindamycin group; n = 10), IFN-α formulation treatment (IFN-α group; n = 14), and combined clindamycin and IFN-α formulation treatment (combination group; n = 16). In the clindamycin group, clindamycin 5 mg/kg was administered via the oral cavity twice per day, beginning 4 days before treatment and ending 3 days after periodontal treatment (a total of 7 days), in accordance with the guidelines of the American Animal Hospital Association^57^. In the IFN-α group, 2.75 g of IFN-α formulation (InterBerryα^***®***^) was applied to the gingival margin of all teeth, 10 times total over 35 days after periodontal treatment. The combination group received both clindamycin and IFN-α formulation treatments, in accordance with the methods described above. Oral swab specimens were collected from the gingival margin of the maxillary right or left canine and fourth premolar using a micro brush (Microapplicator fine, FEED Corporation, Yokohama, Japan), as described previously^24^, several days before and 5 weeks after periodontal treatment.

### Evaluation of periodontal conditions before and after clinical treatment

Periodontal scores were determined by assessment of the gingival margin of the maxillary right or left canine and fourth maxillary premolar, several days before and 5 weeks after periodontal treatment, using a modified version of a previously described method^13^. For each dog, gingival scores of the maxillary right or left canine and fourth premolar were evaluated visually as follows: (1) no significant findings; (2) mild periodontal disease—gingival swelling, gingival regression, and halitosis; (3) moderate periodontal disease—exposure of root, spontaneous bleeding, and tooth loss; and (4) severe periodontal disease—furcation involvement and fistula formation.

### Detection of *P. gulae* and *fimA* genotypes in clinical samples

Distributions of *P. gulae* and *fimA* genotypes were determined using previously developed PCR-based methods^8,25^; Table 3 lists the PCR primers used in this study. Bacterial DNA was extracted from each oral specimen using a Gentra Puregene Yeast/Bact. Kit B (Qiagen, Hilden, Germany). PCR analysis was then performed using universal primer sets targeting 16 S rRNA genes to confirm that bacterial DNA was successfully extracted; subsequently, *P. gulae* and *fimA* genotypes were determined using respective specific primer sets^8,24,25^. Amplification reactions were performed using a total volume of 20 µl with 1 µl of template solution and Ex *Taq* DNA Polymerase (Takara Bio. Inc., Otsu, Japan) with the following cycling parameters: initial denaturation at 95 °C for 4 min; 30 cycles of 95 °C for 30 s, 60 °C for 30 s, and 72 °C for 30 s; and final extension at 72 °C for 7 min. Amplification reactions were performed in an iCycler thermal cycler (Bio-Rad). The resulting products were separated by electrophoresis on a 1.5% agarose gel-Tris-acetate-EDTA buffer. The gel was stained with 0.5 μg/ml ethidium bromide and photographed under ultraviolet illumination.

**Table 3:** Polymerase chain reaction primers used in the present study.

Specific primer set	Sequence (5′-3′)	Size (bp)	References
**Universal primer**			
PA	AGA GTT TGA TCC TGG CTC AG	315	^61^
PD	GTA TTA CCG CGG CTG CTG		
Detection of *P. gulae*	TTG CTT GGT TGC ATG ATC GG	314	^24^
	GCT TAT TCT TAC GGT ACA TTC ACA		
**Specification of** ***fimA*** **type**			
Type A *fimA*			
Pgfim-AF	TGA GAA TAT CAA ATG TGG TGC AGG CTC ACG	190	^25^
Pgfim-AR	CTT GCC TGC CTT CAA AAC GAT TGC TTT TGG		
Type B *fimA*			
Pgfim-BF	TAA GAT TGA AGT GAA GAT GAG GGA TTC TTA TGT	357	^25^
Pgfim-BR	ATT TCC TCA GAA CTC AAA GGA GTA CCA TCA		
Type C *fimA*			
Pgfim-CF	CGA TTA TGA CCT TGT CGG TAA GAG CTT GGA	631	^8^
Pgfim-CR	TGT GGC TTC GTT GTC GCA GAA TCC GGC ATG		

### Statistical analysis

Statistical analyses were conducted using GraphPad Prism 6 (GraphPad Software Inc., La Jolla, CA, USA). Intergroup differences were estimated using analysis of variance with Bonferroni correction. Differences were considered statistically significant at *P* < 0.05.

## Supplementary information

Supplementary figures.

## Data Availability

The data are not available for public access because of patient privacy concerns, but are available from the corresponding author on reasonable request.
