# Author Correction: Inhibition of *Porphyromonas gulae* and periodontal disease in dogs by a combination of clindamycin and interferon alpha

**DOI:** 10.1038/s41598-020-63861-4

**Published:** 2020-04-24

**Authors:** Ryota Nomura, Hiroaki Inaba, Hidemi Yasuda, Mitsuyuki Shirai, Yukio Kato, Masaru Murakami, Naoki Iwashita, So Shirahata, Sho Yoshida, Saaya Matayoshi, Junya Yasuda, Nobuaki Arai, Fumitoshi Asai, Michiyo Matsumoto-Nakano, Kazuhiko Nakano

**Affiliations:** 1 Department of Pediatric Dentistry, Osaka University Graduate School of Dentistry, Suita, Osaka Japan; 2 Department of Pediatric Dentistry, Okayama University Graduate School of Medicine, Dentistry and Pharmaceutical Sciences, Okayama, Japan; 3 Yasuda Veterinary Clinic, Meguro, Tokyo Japan; 4 Department of Pharmacology, School of Veterinary Medicine, Azabu University, Sagamihara, Kanagawa Japan; 5 Department of Veterinary Public Health II, School of Veterinary Medicine, Azabu University, Sagamihara, Kanagawa Japan; 6 Department of Molecular Biology, School of Veterinary Medicine, Azabu University, Sagamihara, Kanagawa Japan; 7 Primo Animal Hospital, Sagamihara, Kanagawa Japan

Correction to: *Scientific Reports* 10.1038/s41598-020-59730-9, published online 20 February 2020

This Article contains a typographical error in the Introduction section where,

“In addition, a canine IFN-α formulation (InterBerryα®; Hokusan Co. Ltd., Higashihiroshima, Japan) has been commercially available as a pharmaceutical agent for periodontal treatment in animals since 2014.”

should read:

“In addition, a canine IFN-α formulation (InterBerryα®; Hokusan Co. Ltd., Kitahiroshima, Japan) has been commercially available as a pharmaceutical agent for periodontal treatment in animals since 2014.”

