# Supplementary material for: Inhibition of Porphyromonas gulae and periodontal disease in dogs by a combination of clindamycin and interferon alpha
Source: Sci Rep. 2020 Feb 20;10:3113. doi: 10.1038/s41598-020-59730-9 (PMC7033253; doi:10.1038/s41598-020-59730-9)
Supplement: Supplementary file 1 — Supplementary figures. [file 41598_2020_59730_MOESM1_ESM.pdf]

Inhibition of *Porphyromonas gulae* and periodontal diseases in dogs by a combination of clindamycin and interferon alpha

Ryota Nomura, Hiroaki Inaba, Hidemi Yasuda, Mitsuyuki Shirai, Yukio Kato, Masaru Murakami, Naoki Iwashita, So Shirahata, Sho Yoshida, Saaya Matayoshi, Junya Yasuda, Nobuaki Arai, Fumitoshi Asai, Michiyo Matsumoto-Nakano, Kazuhiko Nakano

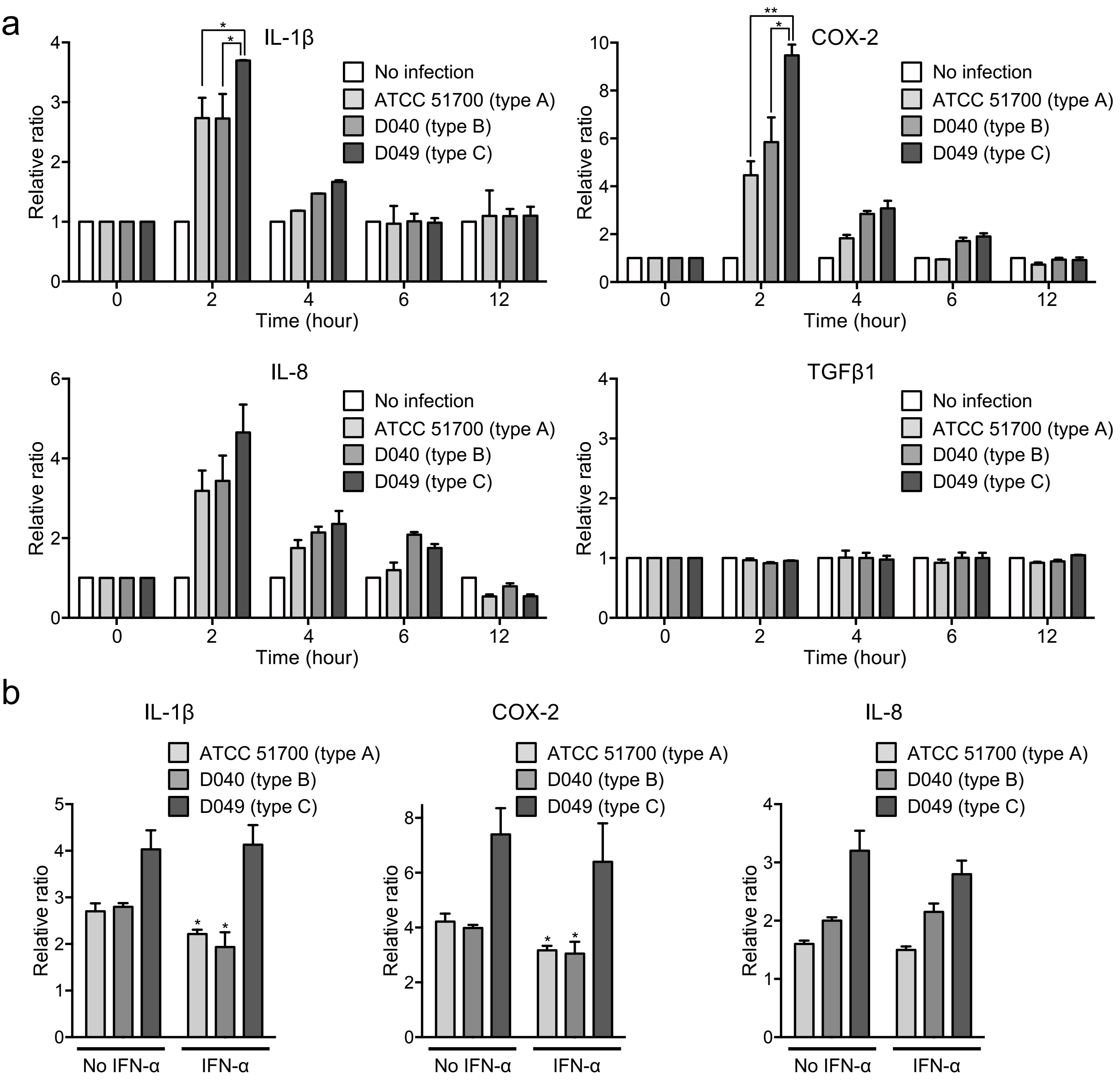

Supplementary Figure 1 Proinflammatory mRNA expression from Ca9-22 cells infected with *P. gulae* strains. (a) Relative ratio of proinflammatory mRNA expression at multiple time points. Proinflammatory mRNA expression before *P. gulae* infection (0 h) was defined as the baseline. There were significant differences as determined by using analysis of variance with Bonferroni correction (**P* < 0.05 and ***P* < 0.01). (b) Relative ratio of mRNA expression in the presence of interferon alpha (IFN-α) formulation. Proinflammatory mRNA expression before *P. gulae* infection (0 h) was defined as the baseline. There were significant differences, relative to no IFN-α formulation, upon infection with each *P. gulae* strain, as determined by using analysis of variance with Bonferroni correction (**P* < 0.05).

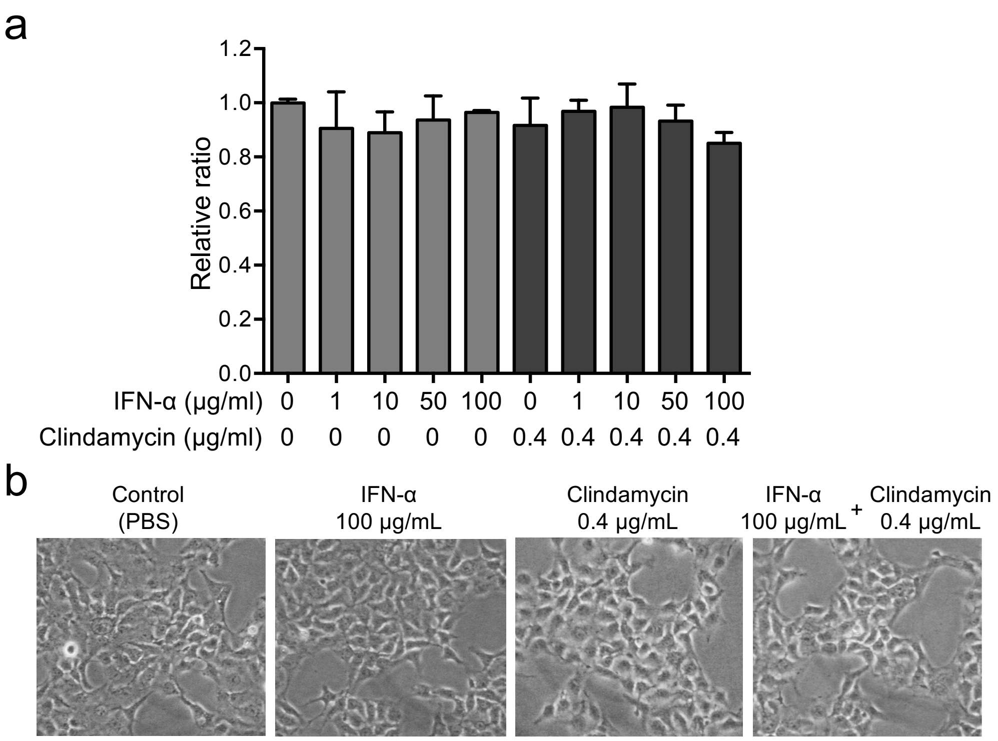

Supplementary Figure 2. Effects of IFN-α formulation and clindamycin on Ca9-22 gingival epithelial cells. (a) Relative ratio of cell growth. The gingival epithelial cell growth without IFN-α formulation and clindamycin was defined as the baseline. (b) Representative images of the gingival epithelial cells with or without IFN-α formulation and clindamycin.
